# The Effect of Integrated Care After Discharge From Hospitals on Outcomes Among Korean Older Adults

**DOI:** 10.34172/ijhpm.2023.7997

**Published:** 2024-01-14

**Authors:** Jae Woo Choi, Ae Jung Yoo

**Affiliations:** Community Care Research Center, Health Insurance Research Institute, National Health Insurance Service, Gangwon, South Korea

**Keywords:** Integrated Care, Discharged Patients, Older Adults, Outcomes, South Korea

## Abstract

**Background:** The Korean government implemented a pilot project for integrated care of older adults in August 2019. the pilot project of integrated care provided housekeeping support, nutrition support, movement assistance, home repair, consultations and education for healthy lifestyle, and some home-based primary care to older patients discharged from hospitals. This study investigated the outcomes of this project among older adults who participated in it after discharge from the hospital.

**Methods:** This study combined the data from the pilot project survey with that from the National Health Insurance Service. The participants comprised 1,895 older adults who participated in the pilot project between August 01, 2019 and April 30, 2022. For comparison, 7,145 older adults who lived in regions where no pilot project were selected as the matched group using propensity score matching. The length of home stay, total expenses of national health insurance and long-term care insurance, emergency visits, and hospital readmission for the same disease were measured, till July 31, 2022. Statistical analysis was performed through difference-in-differences analysis using a generalized estimating equation and the Cox proportional hazards model.

**Results:** The results indicated an increase of 35.2 days (95% confidence interval [CI] 30.7, 39.8) in length of home stay over an average observation period of 550.5 days and a reduction of 6,960 USD (95% CI: -7,924, -5,996) in total expenses for participants compared to the controls. The odds ratio of emergency visits of the pilot project participants was 0.56 (95% CI 0.48, 0.65) compared with the controls. The hazard ratio for hospital readmission for the same disease after hospital discharge was 3.53 (95% CI 2.98, 4.19) times higher in project participants than that in the controls.

**Conclusion:** The pilot project for integrated care has resulted in an increased length of home stay and hospital readmission and reduced total expenses and emergency visits among older patients discharged from hospitals. The integrated care after discharge from hospitals can help older adults to continue living in the place where they lived, and improved collaboration between clinics and hospitals is required to prevent readmissions.

## Background

**Key Messages**

**Implications for policy makers**

- The pilot project for integrated care increased the length of home stay and reduced the total expenses and emergency visits for older patients discharged from hospitals indicating a positive outcome.
- The project increased hospital readmissions for the same disease in older patients discharged from medical institutions and highlights the need for collaboration among healthcare providers.
- Close collaboration between primary care clinics, hospitals, and local governments is required to further improve the outcome of integrated care of older patients discharged from hospitals.

**Implications for the public**

The pilot project of integrated care in the community aims to support aging in place and reduce the financial expenses of care for older adults in Korea. Our study showed an increase in length of home stay and a reduction in total expenses and emergency visits in participants compared with that in the control group. Older adults need to consider registering for such interventions in future. However, the findings suggested that readmission for the same diseases after discharge from hospitals was significantly increased compared with that in the control group. Improved exchange of patient information between the hospital and primary care following hospital admission allows for quicker follow-up once the patient is discharged. Such follow-ups include the prevention of further illness and readmission to the hospital. Local governments need to enable information exchange between hospitals and primary care and provide prompt support to patients discharged from hospitals through integrated care.

Discharge of patients from the hospital is a critical stage in patient care, especially for older adults because they are more frail or at risk of functional decline.^1,2^ Older adults who have mobility limitation, loss of independence, undernutrition, poor compliance for advisable lifestyle, and unstable chronic disease after discharge from hospitals have higher risk for readmission.^3-6^ The readmissions after discharge from hospitals are associated with higher comorbidities including diabetes, cardiovascular disease, chronic kidney disease, and have higher risk mortality compared to those who readmitted, suggesting importance of integrated care after discharge from hospitals to prevent readmission.^7^

 Integrated care seeks to better coordinate health and social care around an individual’s needs with a commitment to improve the quality of care and overcome fragmented care through ongoing co-productive partnerships.^8^ The Korean government has implemented a pilot project for integrated care in the community for older adults in 13 of 229 local governments since June 2019. The central government planned this project as a service delivery system centered on local governments who evaluate multiple care needs of participants at one place and provide integrated care services to meet their needs.^9^ The pilot project focused on strengthening the linkage between health and social services and supporting older patients discharged from hospitals.

 In Korea, the pilot project of integrated care in the community aims to support aging in place and reduce the financial expenses of care for older adults. The proportion of the elderly population and financial expenditure for social insurance are rapidly increasing, and a policy shift from facility-based care to integrated care in the community is required in Korea.^10^ Korea recently completed the first phase of pilot project from June 2019 to December 2022, and the second phase of the pilot project is scheduled from July 2023 to December 2025. This pilot project is being implemented in some of the local governments across the country, and the Korean government plans to promote the system for integrated care for all local governments (229 regions) nationwide from January 2026. In the first phase of pilot project. Therefore, our evidence for intervention to older patients after discharge support Korean and worldwide policy changes for integrated care.

 Integrated care for patients discharged from medical institutions can help prevent emergency visits.^11^ Moreover, recent studies have shown that patients who cannot benefit from effective home care services tend to have more unplanned emergency department visits, unnecessary hospitalisations, and nursing home placements.^12,13^ Although previous interventions have improved patients’ functional ability and the quality of care as well as decreased hospitalizations, the results are contradictory, and clear beneficial effects have not been found.^14-18^

 This study investigated the length of home stays, total expenses for social insurance, emergency visits, and hospital readmissions among older adults who participated in the pilot project for community care after discharge from medical institutions.

## Methods

###  Data and Study Sample

 This study utilized data from the survey conducted by the local government and from the National Health Insurance Service (NHIS) in Korea. The NHIS has information on sociodemographic variables, death records, the National Health Insurance (NHI) claims data for all visits to medical institutions, and long-term care insurance (LTCI) claims data for all utilizations of long-term care (LTC) services by the Korean population.^19^ As of August 2019, civil servants of 13 Korean local governments have implemented regular surveys (monthly) for information on older adults who participated in the pilot project of integrated care. The survey items include personal information, registration and end dates of the pilot project, and types of utilization services for participants.

 Among the 25 692 individuals initially registered between August 2019 and April 2022, this study excluded those under 65 years of age as of the participation date (n = 1101), those with non-identifying data such as errors in social security number (n = 3134), those who were not linked to the NHIS data (n = 33), those who did not utilize the integrated care services despite being registered (n = 1889), and those who did not agree to the utilization of personal information for the study (n = 72), those with an observation period of less than 30 days from the participation date of the pilot project because it takes about two weeks to a month for pilot project participants to use integrated care services (n = 945), and those who were not discharged at least one year before the utilization of integrated care services among participants because the subjects of our study are older patients who used integrated care services after being discharged from a medical institution (n = 15 906).

 For comparison, older adults who lived in regions where no pilot project was conducted were selected as the control group. This study implemented 1:4 propensity score matching (PSM) to reduce selection bias^20^ and conducted a two-stage PSM: the first stage was at the local government level, and the second was at the individual level because the pilot project was implemented at the regional level, and the provision of integrated care services was relatively affected by the composition of the region’s population and resources. In the first stage of PSM, 52 control regions were chosen from 229 local governments—with an average of approximately 200 000 people in each local government—based on four common characteristics: proportion of older adults, total population, neighborhood deprivation index,^21^ and type of region (metropolitan, local city, rural area, and urban-rural consolidated city). In the second stage of PSM, data on older adults who lived in each of the four matched control regions were extracted and matched with participants having similar characteristics (age, sex, household income, Charlson Comorbidity Index [CCI], disability, mental illness, living status, and LTCI grade). After completing the two-stage PSM, those with different characteristics than the control group were excluded from the analysis (n = 717). The final study participants comprised 1895 older adults, and the control group comprised 7175 patients discharged from hospitals (Figure).

**Figure:**
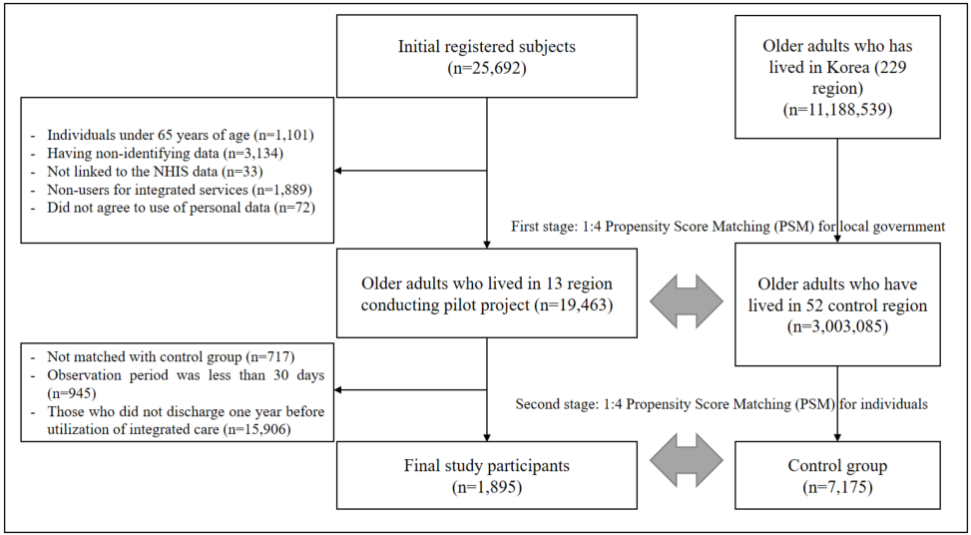
Flow Chart of Study Participants. Abbreviation: NHIS, National Health Insurance Service.

###  Interventions

 Although the forms and the details of integrated services were different by local government, the pilot project of integrated care provided housekeeping support, nutrition support such as lunch box delivery, movement assistance, consultations and education for healthy lifestyle, home repair, LTCI services (home visit care, home visit nursing, home visit bathing, day and night care, short-term care, and provision of assistive devices) for eligible, and home-based primary care (HBPC) for homebound to older patients discharged from hospitals. On average, the HBPC services was provided to patients once in a month or 2 months until termination of services. The HBPC services was commonly carried out with education and counseling on healthy lifestyle and arrangement of medications. The injections for pain, intravenous nutrition therapy, rehabilitation and exercise therapy were provided in some areas, consisting of nurses and physical therapists in addition to doctors as a team.^22^

###  Measurements

 The outcomes of this study were length of home stay, total expenses of social insurance (NHI and LTCI), emergency visits, and readmissions for the same disease after hospital discharge. Length of home stay was calculated by eliminating the period of admission to medical institutions or LTC facilities during the entire observation period. Total expenses of social insurance were estimated by adding the insurer’s and patient’s co-payments, and the monetary units were converted to US dollar (as of February 14, 2023). In the case of LTCI, if there were differences between the maintenance period of LTCI eligibility before and after the observation period, LTCI expenses were estimated by applying the same to the shorter maintenance period of LTCI eligibility of the pre- or post-observation period. Emergency visits were measured using the claims records for benefits of emergency management. Finally, hospital readmission was measured in case of readmission after participation in the pilot project for the same disease at the time of discharge from the medical institutions within one year before the participation date of the pilot project.

 All matched variables in this study were measured based on the date or year of participation, and their characteristics were defined as follows: CCI is the summation of weighted scores assigned to major health conditions^23^ and was estimated using claim records for one year before the participation date in the pilot project. Household income was classified as Q5 (81st–100th percentile, highest), Q4 (61st–80th percentile), Q3 (41st–60th percentile), Q2 (21st–40th percentile), and Q1 (<20th percentile, lowest).

 Mental illness was measured using medical records of psychiatric diseases (International Classification of Diseases, Tenth Revision [ICD-10] codes: F00–F99) for 3 years before the participation date. For LTCI, grades 3–5 and cognition support grades are relatively less severe and home care services are recommended, while grades 1–2 comprise the most severe conditions in which LTC facilities are recommended. To consider the levels of physical function in older adults, we defined grades 1–2, 3–5 and cognition support grade, and non-beneficiaries as LTCI grades. It was measured using the latest records for five years before the participation date. We measured living status using information on the number of household members living together.

###  Statistical Analysis

 For the general characteristics of this study, values were indicated as mean ± standard deviation for continuous variables or as numbers with percentages for categorical variables. The homogeneity test for matching the variables between the participants and control group was conducted using standardized difference, which compares the difference in means in units of the pooled standard deviation and is being increasingly used to compare the balance in baseline covariates between participants who were treated and untreated in a PSM sample.^24^ An absolute value greater than 0.1 of standardized difference denotes a meaningful imbalance in the baseline covariate.^25^

 The difference-in-differences (DID) methodology was used for the length of home stay, total expenses, and emergency visits to compare pre- and post-reform changes between the participants and control group.^26^ Each observation period from the date of participation to July 31, 2022, was set as the post-observation period for the study participants. If the service utilization of the pilot project was terminated or the participant died, the observation period from the date of participation was assigned until that date. For the same pre-post comparison, the post-observation period was the same as before, based on the date of registration. To observe changes within the same observation period, the same participation date and the pre- and post-observation periods were allocated to each matched control group.

 As this study compared the before-after values for the same participant, it was necessary to consider the correlation within the participants. To validate the statistical significance of the difference in before-after results by participation in the pilot project, we performed McNemar’s test for categorical variables and paired t-test for continuous variables. DID analysis was conducted using generalized estimation equations to consider the characteristics of the repeated measurement data. The results of the DID were analyzed by the interaction term value between the variable for pre- and post-pilot project period and the variable for the participants of the pilot project. If the outcome was a continuous variable, we calculated the yearly (365 days) average value per individual by standardizing the observation period, as the observation periods differed for the participants (Supplementary file 1, Figure S1).

 As events regarding readmission to hospitals were not suitable for pre-post comparative analysis, the Cox proportional hazards regression model was utilized using only the post-observation period. This study measured the hazard ratios (HR) and 95% confidence intervals (CIs) for events related to hospital readmission (Figure S2). Proportional hazard assumptions were assessed statistically and satisfied for all models. SAS software (version 9.4; SAS Institute Inc., Cary, NC, USA) was used for the extraction and statistical analyses of all data.

## Results

 This study included 1895 (20.9%) older adults who participated in the pilot project after discharge from hospitals and 7175 (79.1%) in the matched control group. The total average observation period was 550.5 days (standard deviation: 386.3 days), including the pre- and post-observation periods of 275.3 days each. Table 1 presents the general characteristics of the study participants. The average age of the participants was 78.1 years, and 67.3% were women. Older adults with low household income levels and CCI scores of 1 or higher comprised 35.5% and 91.4% of the participants, respectively. Older adults with mental illness and disability comprised 44.4% and 30.2% of participants, respectively, and those with grades 1–2, and 3-5 and cognition support grades comprised 1.2% and 16.4% of the participants, respectively. Among the participants, 54.4% were living alone. There were no statistically significant differences in the variables between the participants and the control group.

**Table 1:** General Characteristics of Study Subjects

**Variables**	**Pre-PSM**					**Post-PSM**		
	**Older Adults Who Used Integrated Care Services After Discharge From Hospitals**		**Older Adults Who Did Not Use Integrated Care Services After Discharge From Hospitals**		**SD**	**Older Adults Who Did Not Use Integrated Care Services After Discharge From Hospitals**		**SD**
	**No.**	**%**	**No.**	**%**		**No.**	**%**	
Total	1895	20.9	3 003 085	100.0		7175	79.1	
Gender					0.314			-0.064
Men	619	32.7	1 347 429	44.9		2130	29.7	
Women	1276	67.3	1 655 656	55.1		5045	70.3	
Age (mean ± standard deviation)	78.1	6.8	72.6	7.7	1.008	78.3	7.1	-0.022
Household income					0.314			-0.064
Q1 (Lowest)	673	35.5	172 117	5.7		2454	34.2	
Q2	262	13.8	563 815	18.8		1016	14.2	
Q3	229	12.1	489 602	16.3		805	11.2	
Q4	238	12.6	651 630	21.7		839	11.7	
Q5 (Highest)	493	26.0	1 082 021	36.0		2061	28.7	
CCI					0.314			-0.064
0	163	8.6	850 742	28.3		576	8.0	
1	253	13.4	698 596	23.3		991	13.8	
2	279	14.7	526 164	17.5		1133	15.8	
3	304	16.0	356 147	11.9		1116	15.6	
4	252	13.3	230 398	7.7		963	13.4	
5	198	10.4	141 107	4.7		807	11.2	
≥6	446	23.5	199 931	6.7		1589	22.1	
Mental illness					0.445			0.014
Yes	841	44.4	630 002	21.0		3133	43.7	
No	1054	55.6	2 373 083	79.0		4042	56.3	
Disability					0.385			0.016
Yes	573	30.2	418 805	13.9		2116	29.5	
No	1322	69.8	2 584 280	86.1		5059	70.5	
Grade of LTCI					0.385			0.016
1-2 grade	23	1.2	50 986	1.7		57	0.8	
3-5 grade and cognition support grade	311	16.4	197 005	6.6		1087	15.1	
None	1561	82.4	2 755 094	91.7		6031	84.1	
Living alone					0.706			-0.050
Yes	1031	54.4	736 471	24.5		4081	56.9	
No	864	45.6	2 251 200	75.0		3094	43.1	

Abbreviations: PSM, propensity score matching; SD, standardized difference; CCI, Charlson Comorbidity Index; LTCI, long-term care insurance.

Table 2 shows the results for changes in the length of home stay and total expenses by the utilization of integrated care after discharge from hospitals. For older adults who participated in the pilot project, the yearly average length of home stay at pre- and post-participation were 303.3 and 335.9 days, respectively. The values showed a statistically significant increase of 32.6 days (95% CI: 28.4, 36.7) after participation in the pilot project, and that of the control group showed a decrease of 2.7 days (95% CI: -4.4, -0.9). The results of the DID analysis showed that the length of home stay for older adults who participated in the pilot project significantly increased by 35.2 days (95% CI: 30.7, 39.8) compared to that for the control group.

**Table 2:** The Results for Changes in Length of Home Stay and Total Expenses by the Utilization of Integrated Care After Discharge From Hospitals

**Outcomes**	**Subjects**	**N**	**Pre-participation**		**Post-participation**		**Pre-post Differences**		**DID**	
			**Mean**	**STD**	**Mean**	**STD**	**Mean (95% CI)**	***P*** ** Value**	**Mean (95% CI)**	***P*** ** Value**
Length of home stay	Participants	1895	303.3	81.3	335.9	68.2	32.6 (28.4, 36.7)	<.001	35.2 (30.7, 39.8)	<.001
	Control group	7175	324.7	135.4	322.0	127.8	-2.7 (-4.4, -0.9)	.003		
Total expenses	Participants	1895	17 134	16 930	11 050	16 743	-6084 (-6957, -5211)	<.001	-6960 (-7924, -5996)	<.001
	Control group	7175	8915	12 550	9791	20 431	876 (482, 1270)	<.001		

Abbreviations: STD, standard deviation; DID, difference-in-differences; CI, confidence interval. Unit: days, US dollar.

 The yearly average total expenses per individual pre- and post-registration for older adults who participated in the pilot project were US$ 17 134 and US$ 11 050, respectively. While the average total expenses for project participants showed a statistically significant decrease of US$ 6084 (95% CI: -6957, -5211) post-participation, that for the control group showed an increase of US$ 876 (95% CI: 482, 1270). The DID analysis showed that the total expenses of the pilot project participants were significantly reduced by US$ 6960 (95% CI: -7924, -5996) compared with that of the control group.

Table 3 shows the results of changes in emergency visits by the utilization of integrated care after discharge from hospitals. For project participants, the proportion of emergency visits pre- and post-participation were 38.2% and 20.5%, respectively. While the emergency visits for project participants showed a statistically significant decrease of 17.7%p post-participation, that for the control group showed a decrease of 3.7%p. The DID analysis showed that the odds ratio of emergency visits of the pilot project participants was 0.56 (95% CI: 0.48, 0.65) compared with the control group.

**Table 3:** The Results of Changes in Emergency Visits by the Utilization of Integrated Care After Discharge From Hospitals

**Subjects**	**N**	**Pre-participation**		**Post-participation**		**Pre-post differences**		**DID**	
		**No.**	**%**	**No.**	**%**	**%p**	***P*** ** Value**	**OR (95% CI)**	***P*** ** Value**
Participants	1895	724	38.2	389	20.5	-17.7	<.001	0.56 (0.48-0.65)	<.001
Control group	7175	1230	17.1	964	13.4	-3.7	<.001		

Abbreviations: DID, difference-in-differences; OR, odds ratio; CI, confidence interval.

Table 4 indicates the results for readmissions due to the same disease by the utilization of integrated care after discharge from hospitals. During the post-observation period, the number of hospital readmissions for the same diseases after hospital discharge among the project participants was 251, while that among the control group was 275. The HR for hospital readmission for the same disease after hospital discharge for project participants was 3.53 times (95% CI: 2.98, 4.19) higher than that for the control group.

**Table 4:** The Results for Readmissions due to Same Disease by the Utilization of Integrated Care After Discharge From Hospitals

**Subjects**	**N**	**Events**	**HR**	**95% CI**	***P*** ** Value**
Control group	7175	275	1.00	2.98-4.19	<.001
Participants	1895	251	3.53		

Abbreviations: HR, hazard ratio; CI, confidence interval.

## Discussion

 This study evaluated the length of home stay, total expenses of NHI, emergency visits, and hospital readmission among older adults who participated in the pilot project after discharge from hospitals. The results of the DID analysis showed an increase in length of home stay and a reduction in total expenses and emergency visits in project participants compared with that in the control group. However, the findings suggest that readmission for the same diseases after discharge from hospitals was significantly increased compared with that in the control group.

 An increase in length of home stay indicates a decrease in length of hospitalization or institutionalization. A few previous studies demonstrated lower hospital admission and length of hospital stay after implementation of integrated care,^27-29^ while other studies reported no significant differences in hospital admission and length of hospital stay between the intervention and control groups.^30-32^ Our study provides evidence that integrated care has contributed to increasing length of home stay and it is believed that the provision of post-discharge integrated care in Korea has had a positive impact on reducing unnecessary hospitalization of the elderly.^33^

 Previous studies on changes in costs due to integrated care in the community have shown inconsistent results.^34-36^ This study revealed cost savings through the pilot project, which can be explained based on the results of length of home stay. Korean statistics in 2021 reported that the average monthly expenses of LTC facilities (US$ 1599) were approximately twice as high as those of LTC home services (US$ 852) among LTCI recipients.^37^ The average daily expense of inpatient treatment (US$ 167) for patients aged 65 years or above was approximately four times higher than that of outpatient treatment (US$ 40).^38^ Therefore, it can be inferred that the total expenses decreased because of the reduction in the stay in hospitals or LTC facilities.

 Previous research on changes in emergency visits after implementation of integrated care showed inconsistent results.^30,39-42^ The reduction in emergency visits could mean that integrated care in the community provided an appropriate assistance system for older patients discharged from hospitals, even if they were clinically frail or lived under socially frail conditions. Recent data from over 400 000 emergency visits indicated that utilization of home care reduced the risk of emergency visits in older adults living in socially deprived areas.^43^ Therefore, integrated care in the community could promote efficient healthcare system utilization not only because of its clinical aspects but also by alleviating the effects of living in deprived areas.

 Our findings showed that readmission for the same disease after discharge from hospitals was significantly higher for project participants than that for the control group. No significant difference in readmission was observed in a previous study.^30^ In Korea, medical delivery systems are incomplete. In particular, hospitals and primary care compete within a region instead of having a structure in which patients discharged from hospitals are referred to primary medical institutions. An incomplete medical delivery system would result in insufficient communication between inpatient and outpatient physicians. Improved exchange of patient information between the hospital and primary care following hospital admission has allowed for quicker follow-up once the patient has been discharged. Such follow-ups include prevention of further illness and thus readmission to the hospital. Local governments need to enable information exchange between hospitals and primary care and provide prompt support for patients discharged from hospitals through the integrated care until the medical delivery system is improved.

 Although this study revealed crucial findings, it had a few limitations. First, the pilot project for integrated care in Korea provided services based on the needs of participants. Thus, the quantity and type of services for each participant were different, making it difficult to verify a single service or combination of services that contributed to the project outcomes. Future studies are required to assess the effectiveness of the pilot project by developing a standardized integrated care model. Second, as budget-based expenses for the integrated care services provided by local governments could not be identified, therefore, it was excluded from the calculation of total expenses. Third, different concurrent explanations may explain the limited number of studies that produce significant statistical differences. One plausible explanation, known as the “omitted variable bias,” notes that outcome measures such as hospital readmissions and length of stay are moderated by other confounding variables that are usually not captured in large-sample studies. Conversely, the same outcomes cannot be easily observed in smaller sample in-depth studies, due to the impact of sample size in hypothesis testing. Another explanation is the “timeframe gap,” whereby studies do not analyse effects over a sufficiently long period to observe changes that accrue over lengthier time frames.^44^ Fourth, although a control group with similar characteristics to participants in pilot project was selected by PSM, there is a slight difference between the average values for outcomes of the participants and the control group before participating in the pilot project, suggesting necessity to consider the difference when the results are interpreted.

 In conclusion, the present study is the first to explore the effect of integrated care after hospital discharge on outcomes among Korean older adults. Integrated care through cooperation between medical institutions and the local government has a positive effect on patients discharged from hospitals. Close-knit collaboration between patient-centered primary care clinics, hospitals, and local governments is required for improvement in integrated care of older patients discharged from hospitals.

## Ethical issues

 This study was approved by the institutional review board of the National Health Insurance Service (Approval number: 2022-HR-03-024).

## Competing interests

 Authors declare that they have no competing interests.

## Funding

 This work was supported by Ministry of health and welfare in Korea [Grant/Award Number: 00228145200]. The funder had no role in the design, methods, data collection, analysis or preparation of the manuscript.

## Supplementary files

Supplementary file 1 contains Figures S1 and S2.
