# Supplementary material for: The Effect of Integrated Care After Discharge From Hospitals on Outcomes Among Korean Older Adults
Source: Int J Health Policy Manag. 2024 Jan 14;13:7997. doi: 10.34172/ijhpm.2023.7997 (PMC11607591; doi:10.34172/ijhpm.2023.7997)

Article title: The Effect of Integrated Care After Discharge From Hospitals on Outcomes Among Korean Older Adults

Journal name: International Journal of Health Policy and Management (IJHPM)

Authors' information: Jae Woo Choi, Ae Jung Yoo*

Community Care Research Center, Health Insurance Research Institute, National Health Insurance Service, Gangwon, South Korea.

***Correspondence to:** Ae Jung Yoo; Email: aejungyoo@gmail.com

Citation: Choi JW, Yoo AJ. The effect of integrated care after discharge from hospitals on outcomes among Korean older adults. Int J Health Policy Manag. 2024;13:7997. doi:10.34172/ijhpm.2023.7997

Supplementary file 1

LEGENDS

Supplementary Figure 1. Diagram for difference-in-differences analyses

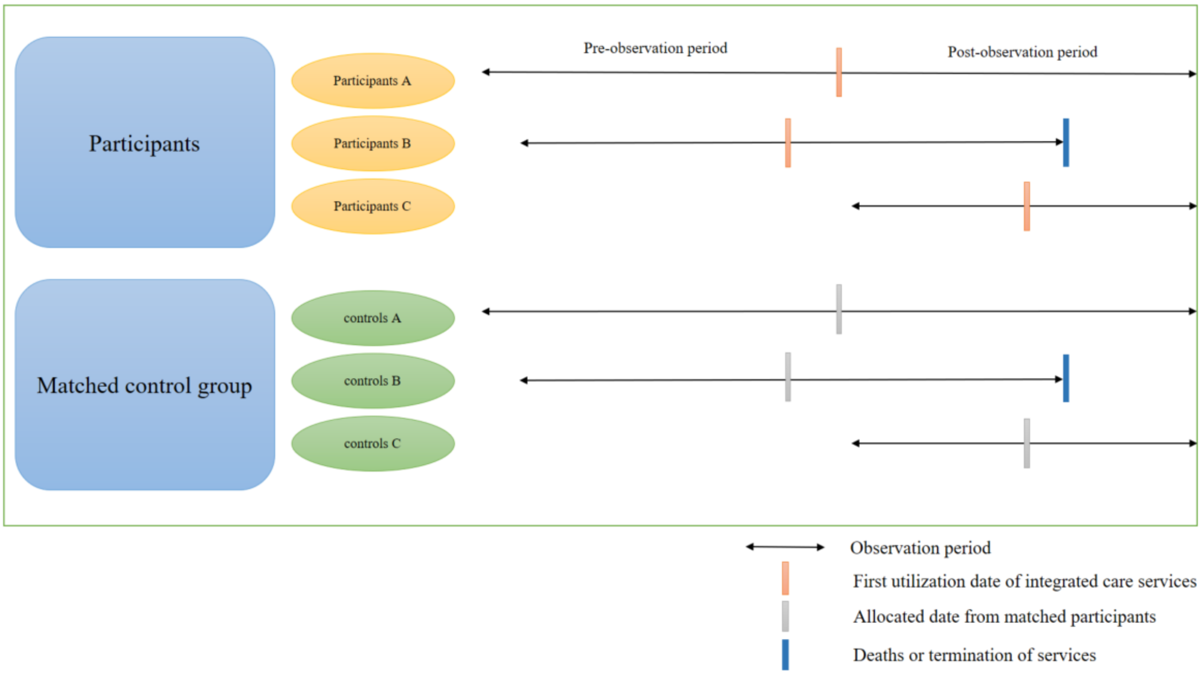

Supplementary Figure 2. Diagram for Cox proportional hazard model

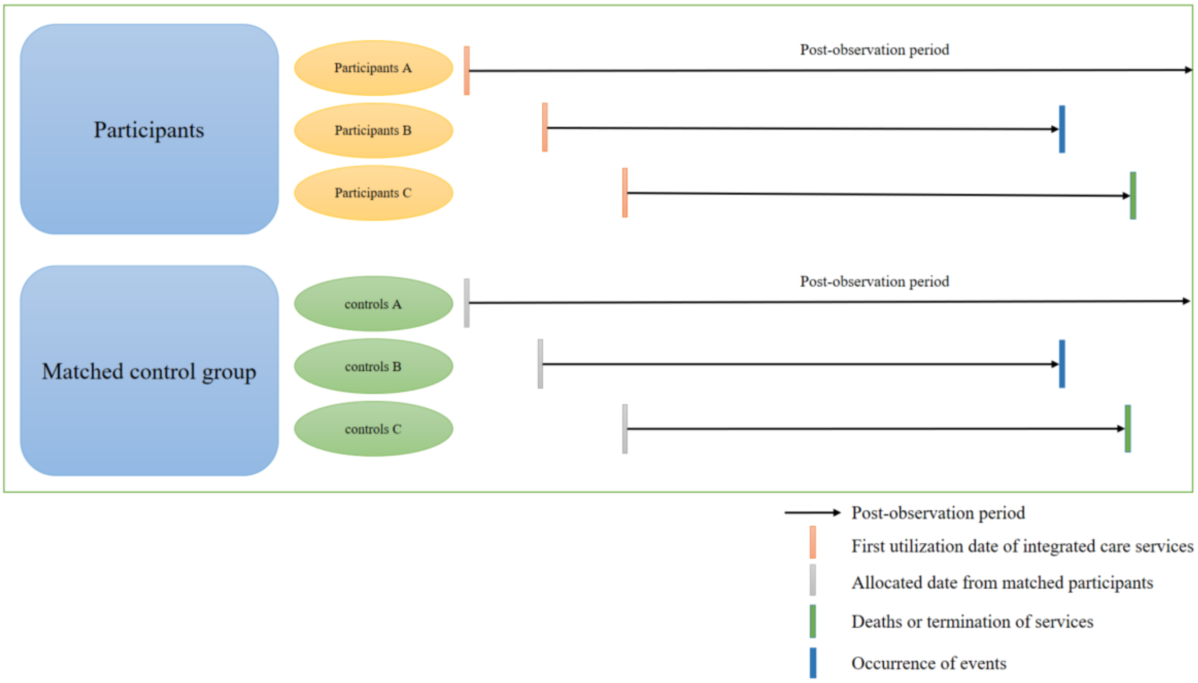

Supplement: Supplementary file 1 — contains Figures S1 and S2. [file ijhpm-13-7997-s001.pdf]
